# Critically Ill Patients: Family Experiences of Interfacility Transfers From Rural to Urban Centers and Impact on Family Relationships

**DOI:** 10.1177/10748407221124254

**Published:** 2022-09-29

**Authors:** Margie Burns, Jill Bally, Meridith Burles, Shelley Peacock

**Affiliations:** 1 University of Prince Edward Island, Charlottetown, Canada; 2 University of Saskatchewan, Saskatoon, Canada

**Keywords:** life shortening illness, family/participant group, family caregivers, qualitative, research methodology/design, phenomenology

## Abstract

A critical illness event is intensely stressful for family members and can lead to negative psychological, emotional, social and financial consequences. In geographically rural areas, critically ill patients may require an interfacility transfer to an urban centre for advanced critical care services. In this context, research suggests that these family members from rural areas experience additional burdens, yet little is known about these experiences. An interpretive phenomenological approach was used to explore lived experiences of family members from rural areas whose critically ill relative undergoes an interfacility transfer to an urban centre for advanced critical care services. Participants described feelings of vulnerability in the urban centre, the need to protect the critically ill patient and other relatives, maintaining responsibilities at home, navigating family relationships, and a loss of connection during the transfer window. These findings may better position nurses to address family members’ stress and anxiety during this experience.

Family members such as spouses, siblings, children, parents, and friends experience intense stress and anxiety when a relative is critically ill and requires care in an intensive care unit (ICU) (DiSabatino Smith & Custard, 2014; Engström & Söderberg, 2004). In some situations of critical illness, specifically in geographically rural areas, the critically ill patient requires advanced care services not available at their local hospital to treat intracranial bleed, spinal cord injury (Burns, 2021; Mackie et al., 2014), major trauma, vascular emergencies, or acute myocardial infarction (Burns, 2021), and an interfacility transfer (IFT) to an urban centre occurs (Blackwell, 2002; Johnson, 1999). Existing literature indicates that an IFT of a critically ill patient to a distant urban centre adds additional stressors for family members (Burns et al., 2018; Kulnik et al., 2019; Mackie et al., 2014) with stress and anxiety noted to be central constructs of the experience (Burns & Petrucka, 2020). Critical care nurses are well positioned to support family members during this experience (Frivold et al., 2015; International Family Nurses Association (IFNA), 2015; Mackie et al., 2014). However, the experiences of family members in rural areas whose critically ill relative undergoes an IFT for advanced critical care services are poorly understood (Burns & Petrucka, 2020; Karlsson et al., 2020). In addition to this limited understanding, Burns and Petrucka (2020) found that healthcare professionals do not view the experiences of family members from rural areas as unique, thereby potentially neglecting their distinct care needs.

Regionalization of health care, where secondary and tertiary services are concentrated in select centers (Kahn et al., 2008), has resulted in the necessity of interfacility transfer (IFT) of critically ill patients from general hospitals to distant, specialized hospitals for advanced critical care services (Blackwell, 2002; Canadian Institute for Health Information [CIHI], 2010, 2016). A critically ill patient’s IFT has been recognized as generating additional stress for family members (Burns et al., 2018; Kulnik et al., 2019; Mackie et al., 2014), although contributing factors are less understood. According to Mackie et al. (2014), family members from rural areas who experience a critically ill relative’s IFT to a distant urban center have needs that are distinct from their counterparts in urban areas. For example, family members from rural areas need support related to being in the unfamiliar surroundings of the urban environment, accommodation needs, transportation needs, and support with the financial burden associated with an IFT to a distant urban center (Mackie et al., 2014).

Within their role, critical care nurses are responsible for ensuring the health care needs of the patient are met and providing comprehensive care to family members (Ågård & Harder, 2007). As such, critical care nurses are in a position to effectively support family members from rural areas who experience significant stress and anxiety during a critically ill relative’s IFT to a distant urban center (Burns & Petrucka, 2020; Mackie et al., 2014). However, critical care nurses must first gain an understanding of the unique experiences of family members from rural areas to ensure appropriate and meaningful care. While there has been increasing interest in the needs of family members of critically ill patients who undergo an IFT to a distant urban center in Australia (Johnson, 1999; Mackie et al., 2014) and Sweden (Karlsson et al., 2020), no studies were located that explore this phenomenon in North America.

The aim of this study was to enhance understanding of the experiences of family members from rural areas whose relative undergoes an IFT for advanced critical care services in Atlantic Canada. The guiding research question was: What is the meaning of the lived experiences of family members from rural areas whose relative undergoes an IFT to an urban tertiary center for advanced critical care services? While this study explored multiple possible experiences for family members from rural areas, this manuscript focuses on experiences within the context of family relationships. Through increased awareness and understanding of these context-specific experiences, nurses can be better attuned to the needs of family members from rural areas and subsequently individualize the nursing care of these individuals (Munhall, 2012).

## Design and Methods

Central to phenomenology is the belief that knowledge arises from human experience and, as such, this methodology aligns with both the philosophy and goals of nursing to inform practice (Annells, 1996; Mackey, 2005; Munhall, 2012). Guided by Munhall’s (1994, 2012) interpretive phenomenological approach, this study understood the context within which a phenomenon was experienced directly influenced meaning and, therefore, within differing contexts, interpretations of experiences diverged and became alternate, individual perspectives of realities (Munhall, 2012). To comprehend an individual’s perspective of a phenomenon, data are processed through the lenses of the four existential life-worlds: corporeality, relationality, spatiality, and temporality (Munhall, 1994, 2012; van Manen, 1990). These existential life-worlds are interconnected and together form the individual’s singular *life-world* that is lived (Munhall, 1994, 2012; van Manen, 1990). The four life-world “existentials” make explicit the context from which meanings of experiences arise, thus enhancing the understanding of multiple possible perspectives of a lived experience (Munhall, 1994, 2012; van Manen, 1990, p. 101). With an embedded focus on the context of nursing practice, this approach is useful in developing nursing knowledge and guiding practice within nurse–patient interactions (Burns & Peacock, 2019; Munhall, 2012).

## Setting and Participants

This study was set in one region of Atlantic Canada. Within this setting, critically ill patients who require specialized critical care not available locally (e.g., neurosurgery, trauma, or cardiovascular services) undergo IFT to a distant urban tertiary care center that is, on average, a journey of 275 kilometers from the originating hospital and includes highways, ferries, and bridges. Definitions of the main concepts within the study are provided in Table 1, including specification of what is meant by rural and urban settings as informed by prior research.

**Table 1.** Definitions of Main Concepts.

Concept	Definition	Citation
Rural	Considered within the context of access to advanced critical care services, as a participant’s “geographic location that necessitates, at times, transfer to an urban centre for advanced critical care services” that are not available locally	Burns & Petrucka (2020, p. 180)
Family member	A “spouse, parent, child, sibling, grandchild, or someone with whom the patient has a similar relationship” (e.g., friend)	Burns & Petrucka (2020, p. 180)
Critical illness	An urgent, life-threatening condition that results in the patient attending the emergency department and then subsequently is admitted to a critical care unit for critical care services not available in a general nursing unit	Burns & Petrucka (2020)
Interfacility transfer	The transfer of a critically ill patient to a tertiary care center offering specialized advanced critical care services not available at the originating center and is more than 150 km away from the originating hospital	Burns & Petrucka (2020); Canadian Institute of Health Information [CIHI] (2010)

Participant recruitment occurred over 14 months and included the time preceding and during the COVID-19 pandemic. Underpinned by philosophical assumptions of the interpretive paradigm, participant recruitment was approached from a relativist ontology where each participant experiences their own unique reality (Denzin & Lincoln, 2018; Munhall, 1994, 2012). Therefore, rather than seeking data saturation, defined as the point when data begin to repeat and no new understandings are emerging during data collection and analysis (Polit & Beck, 2017), participant recruitment continued until a heterogeneous sample with a diversity of experiences were recruited (Peacock et al., 2014). This approach ensured a depth and breadth of experiences were obtained (Munhall, 2012).

While the pandemic was not the focus nor driver of this study, it did influence the findings, as will be described later. Recruitment commenced through advertisements posted in a rural hospital emergency department and critical care family waiting areas, telephone invitation, and snowball sampling. An emergency room staff nurse identified patients who were critically ill and required an IFT to an urban center for advanced critical care services within the last 24 months, but not within the last month. It has been noted that even 27 months after a relative’s critical illness event, family members can describe their experience so precisely, that is, seems the event occurred recently (Ann-Britt et al., 2010); similar time frames for interviews have also effectively been used in more recent studies (Melby et al., 2020). Upon identifying patients who met this criterion, the emergency room nurse accessed the patient’s chart to obtain the next of kin telephone number. Subsequently, the nurse telephoned potential participants and invited them to participate using a script prepared by the first author. If individuals indicated interest in participating, the emergency room nurse provided their preferred mode of communication to the first author. The first author contacted potential participants and assessed if they met the inclusion criteria (see Table 2), and then provided information about the study. If a person chose to participate, a time and location for the first interview was mutually decided.

**Table 2.** Inclusion Criteria.

Criterion	Description
1	Resided in a rural area
2	Were a family member of a critically ill patient who underwent an IFT to an urban center for advanced critical care services
3	Were 18 years of age or older
4	Spoke and understood English
5	Were willing to share their experience
6	At least 1 month had passed since their relative’s IFT event, but not more than 24 months

*Note*. IFT = interfacility transfer.

## Data Collection Tools and Methods

Participants engaged in one to two audio-recorded interviews, 1 to 2 weeks apart. Two interviews were completed prior to the implementation of public health restrictions related to the COVID-19 global pandemic. Interviewing was then paused until public health restrictions permitted the resumption of in-person interviews. Public health guidelines, such as social distancing, were followed for all subsequent interviews. Interviews were conducted by the first author who has experience completing phenomenological interviews. In addition, co-author S.P., who also has experience completing phenomenological interviews, reviewed the audio recording of the first participant interview to verify the appropriate application of an interpretive phenomenological interview approach. Interviewing involved an open-ended interview style (see Table 3); as encouraged by Munhall (2012), interviews were conversational in nature and occurred in a setting where the participant felt most comfortable, predominately in their home. During interviews, participants were encouraged to share other forms of data that would shed light on the meaning of their experiences, such as music that was meaningful and personal journals that participants maintained during that time. A second interview with all but two participants was completed because new understandings of the meaning of experiences can arise in the days following the initial interview (Munhall, 2012).

**Table 3.** Interview Guide.

Interview	Question(s)
To begin interview 1	Can you describe your experience in as much detail as possible when *name* was critically ill and then needed to be transferred to a specialist ICU for advanced care?
To begin interviews 2 and 3 if appropriate (questions for subsequent interviews will emerge from the previous interview[s])	Sometimes in the days after talking about an experience, a person may think more about the experience and additional thoughts or ideas may come to mind. Have you thought about what we talked about the last time we met? If so, what did you think about? Did you talk about what this experience was like for you with another person since our last conversation? If so, would you like to share what you discussed and any thoughts you had afterward? Did you write any notes about your experience since we last met? If so, would you be willing to share them with me? Can you think of any books, music, or movies that you feel resonate with what this experience was like for you? Is there anything else you would like to share?
Other prompts to consider during the interviews if necessary	How did you make sense of . . .? Can you expand on . . .? Help me understand . . .? It was exactly what . . .? Did you have concerns about money or finances during this time? If so, could you describe what these were and what this part of the experience was like for you? If there is a period of silence: Can you tell me what you are thinking about?

*Note*. ICU = intensive care unit.

The first author approached data collection and analysis from the perspective of *unknowing*. In contrast to *knowing*, which embodies a sense of “confidence that has inherent in it a state of closure,” Munhall (1994, p. 63) encourages researchers to set aside their preconceptions when initiating data collection, thereby allowing new understandings of the phenomenon of interest to emerge. However, while Munhall encourages *unknowing*, the researcher is more than an instrument for data collection. As an individual situated within their own life-world, the researcher approaches inquiry from their subjective reality and, as such, unavoidably contributes to the study as a co-constructor of meaning (Munhall, 2012). A researcher journal with personal reflections was kept as a form of data, contributing to a co-created understanding of the meaning of participants’ experiences (Munhall, 2012).

## Data Analysis

Data were analyzed according to Munhall’s (1994, 2012) interpretive phenomenological approach in which the analytic process unfolds as the study progresses and the phenomenon becomes increasingly understood. Thus, the findings of this study include participants’ experiences as described in interviews, as well as elements of the researcher’s interpretation of the meanings of their experiences. Data management and analysis involved uploading data into NVivo 12 (Version 12.6.0) where, using the analysis process described below, data were organized into the four life-worlds for each participant. As each participant’s data were analyzed, a color-coded data display that was numerically coded to each participant was created to facilitate the recognition of similarities and context-specific differences across participant experiences.

Data collection and analysis occurred simultaneously, with preliminary analysis taking place after initial interviews to inform subsequent interviews. As well, the first author maintained a researcher journal to note thoughts and emerging understandings that were revealed during and after participant interviews. Oftentimes, upon completion of a participant interview, the first author would need to pause on the return drive home to document new thoughts that were emerging in relation to the phenomenon under study. Initial interviews were audio-recorded and transcribed by the first author. Subsequent interviews were transcribed by a transcriptionist and subsequently reviewed for accuracy. During the analytic process that followed, significant dwelling with the data, and going back and forth between the whole and the parts (Munhall, 2012) ensued, which involved listening to recorded interviews, reading and rereading transcripts and participant and researcher journals, listening to songs submitted by participants, and memoing in a researcher journal. Researcher notes, media shared by participants, including music and personal journals, and interview transcripts were all processed through the four life-worlds: corporeality, relationality, spatiality, and temporality (Munhall, 1994, 2012). In doing so, meanings of experiences are situated within context, which enhances understanding of participant experiences (Munhall, 2012). It is in this way that concealed and unique, multiple realities as experienced by individuals’ being-in-the-world come to the forefront and understandings are enhanced (Munhall, 1994, 2012).

As each participant’s data were processed, a narrative of experience was produced and shared with the participant to determine if the meaning of their experience of the phenomenon was accurately captured (Munhall, 2012). Ten of the 11 participants indicated that the narrative was accurate; one participant declined to review their narrative. These individual narratives, in part, informed the development of an overarching narrative that captures the many possible interpretations of the meaning of experience as situated in context (Munhall, 2012). In addition, analysis permitted a critique of current practices and considers possibilities for improving nursing care of family members from rural areas whose relative undergoes an IFT to an urban tertiary center for advanced critical care services (Munhall, 2012).

## Rigor

When employing an interpretive phenomenological approach, methodological decisions are made as the study evolves (Munhall, 1994). To enhance rigor, the authors collaborated in the decision-making processes and, when issues arose, they were documented in the researcher journal. In addition, strategies specific to evaluating the rigor of a phenomenological study were adopted (Munhall, 1994). Specifically, the final overarching narrative was shared with two participants who confirmed that it: felt familiar to them (the phenomenological nod), rang true to them (resonancy), was a credible account of the meaning of the experience (reasonableness), captured the full scope of the meaning of the experience (representativeness), and uncovered a deeper understanding of the meaning of the experience (revelations) (Munhall, 1994). The overarching narrative was also shared with the co-authors and individuals unrelated to the study to assess if those who have not experienced this phenomenon ascertained elements within it and subsequently developed a heightened awareness (recognizability) and understanding of the meaning of the experience (raised consciousness). As well, review of the narrative deemed that it was comprehensible and interesting (readability), the results were full and applicable to those who receive care (richness, relevance), ethical (responsibility), elicit a deeper understanding of the experience (revelations), and inspire action (responsiveness) (Munhall, 1994).

## Ethical Considerations

Ethical approvals from the affiliated university and the local health authority where the study was conducted were received prior to initiating the research. At the time of first meeting with participants, the first author provided a letter of invitation and written consent form and discussed important details of the study and answered any questions. Once participants indicated their willingness to participate by providing consent, data collection commenced through audio-taped interviews. Process consent was used for subsequent interviews and confidentiality was maintained through the use of pseudonyms during dissemination.

## Findings

A total of 11 participants engaged in the research. However, due to the relatively small population of the study location, only general demographic data will be presented (see Table 4) to protect participants’ identities. Data sources included 20 participant interviews, three songs identified by participants, personal journals of two participants, and researcher field notes. Findings related to family relationships are presented within context below and supporting data in the form of interview excerpts are offered in Table 5. While experiences of family members from rural areas may overlap with experiences of family members from urban areas (e.g., staying close to the patient), distinguishing experiential features of family members from rural areas are evident, such as a feeling of vulnerability in the urban center and the loss of connection with the critically ill relative during the IFT window.

**Table 4.** Participant Demographic Data.

Characteristic	Participant data
Age range	33–79 years
Relationship to critically ill patient	Spouse, sibling, child, parent, and friend
Range of time between critical illness event and interview	3–24 months
Reasons for patient IFT	Intracranial hemorrhage, major trauma, cardiovascular events, and respiratory failure
Traveled to urban center	9 (by personal car)
Remained at home location	2
Identify as female	9
Identify as male	2

*Note*. IFT = interfacility transfer.

**Table 5.** Existential Life-Worlds, Meanings, and Supporting Data From Participant Interviews.

Life-world	Meanings	Participant interview data
Corporeality	Vulnerability in the urban center	“The homesickness feeling that you have is miserable [crying].”
		Because when I’m away from home I don’t sleep properly and to be honest, it’s a simple thing but my bowels never work good . . . because of my age it made it hard . . . the whole thing because you’re older . . . I felt it bothered me more that I’d have been able to handle it different if I was younger
	Feeling of peace	“Once I got to the hospital I was like a big [deep sigh] . . . [I thought] ‘Oh, thank god I’m not still driving [in the snowstorm],’ because I was so stressed driving.”
		“[Describing the feeling when they received notification that the critically ill family member arrived safely at the urban centre].” “That was quite a relief. Then it was like, ‘Now I can go to bed.’”
Relationality	Need to protect	“And it’s funny how you feel like you have to see somebody before they go for surgery, but you just wanna’ . . . you wanna’ say goodbye. I don’t know if that’s ‘cause—I guess you’re afraid that if something happens in the OR and they come out and they’re not able to talk to you and stuff? . . . I wanted my face to be the last face she saw . . . I wanted [her] to know that she had people there too, I didn’t want her going in scared.”
		“They always made a point of telling him which was nice because if you couldn’t get in because, [of COVID-19 restrictions] . . . just so they won’t be alone, know that somebody cares.”
	Maintaining responsibilities	“I would get up at six o’clock in the morning to head to [the urban centre where her critically ill partner was], I would stop [on the way to the urban centre] to see my Mom and . . . I was cleaning her house for her because she was very sick from the radiation [treatments]. So, I’d go and clean her house for her . . . then I would head down to [the urban centre] and then I would spend Sunday, Monday, Tuesday, come back here [home] Wednesday . . . do payroll Thursday [for her partner’s company], do whatever I needed to do here with [her company] and then Friday was their payroll then . . . go to see my Mom, to go to [the urban centre]. It was just a constant revolving, that’s what I did.”
		“[Critically ill sister] went into the hospital [at the urban centre] . . . the next day . . . my brother went back in the hospital [at the local hospital] . . . he had taken two strokes. So, it was like . . . just run ragged eh, like just running back and forth . . . he was single so I had to make sure . . . his bills, he gave me all his financial stuff to take care of, and we had to do this and do that to make sure he had everything he needed before he got home. So yeah, I was running for both thinking. Everybody said, ‘you’re gonna be run ragged.’ I said, ‘you know what I’m just doing what I gotta do for him to get him back on his feet.’ I said ‘Because if not what would happen?”
	Supported by health care providers	I felt like the majority of the nurses that we had were very caring and were very supportive. Even in conversations, trying to make connections of some sort . . . any nurse that came in was like, “Oh, where are you from and what do you do?” And, “Oh, well I know somebody from there,” some of those connections stuff that you’re still trying to make and I still feel like, even though you’re in a different province, I still feel like people are there to support you [Husband of participant] has dementia besides, so he can’t always express himself . . . I was just scared that he would answer the questions wrong and they wouldn’t understand [so I wanted to] be there to make sure the questions were answered properly . . . It’s very important to have the right information when you’re going to have a surgery The nurses were the big reason why we realized that, “Okay, we can take a break and they’re gonna take care of him. He’s going to be okay, he’s in good hands.” We really got to know them personally and you just build a trust with them. . . If they feel like you’ve been here too long or you need to get outside or you need to go take a nap, they’ll tell you, “Go outside. Don’t be scared to go outside.” So, it was really them that kind of made me feel like we could take those breaks. After you do it once or twice you realize, “Okay, this is gonna be okay. They’re gonna call if something happens.”
	Navigating relationships	“If I was by myself and had to go over, it would’ve been a different experience . . . without somebody with me to talk to . . . when you can’t go in the ambulance and as old as we are . . . it’s not ideal for me to be going to [city] myself driving”
		“[The use of Facebook was] a bad thing for mental health you know? And I think that people who rely so much on the social media side of things and they don’t get those, the responses that they expect can be very detrimental mentally.”
	Loss of connection	“I’d say the ambulance drive was really the longest part because you have no contact with anybody, and you don’t know what’s going on . . . it’s an unknown. . .You just wanna’ know . . . I’d say that was the toughest part was the drive up, him going and me not being able to have some kind of control I guess . . . Being able to call really.”
		“[During the transfer window] you had nobody [to contact]. There was just that void in there that’s like there’s no way of finding out. The ambulance guys didn’t give me their phone number so I couldn’t phone them. But you’ve got that space, them hours that are just- that you can let everything bad go through your mind, everything that could possibly happen. And it was a foggy night so I was concerned about that too”
		“You feel like you’re bothering everybody . . . they gotta look after the patients so can’t be talking on the phone and looking after them at the same time”
Spatiality	Pulling patient and family close	“I don’t have a lot of memory of that particular, that drive over . . . I was just consumed with thought and what was I gonna’ find when I got to [city]? Because I wasn’t getting phone calls from [city]”
		“[When sharing difficult news over the telephone] It was really hard . . . both of us were crying. Because it’s different when you’re talking to him and you can’t see his face, you can’t hug him, you can’t do anything. I knew he was just devastated . . . he didn’t see it coming”
	Home and away	“[When asked why they wanted to be home] Because I wanted to be back home, back to normal”
		“You miss home and you miss your routine and you miss your friends and your family and for me, it’s my husband and my son. He’s only five, so he kind of understands that [her younger child] is sick and has to go but he doesn’t understand why he can’t be there too. I’ve tried that, we have tried taking him over but it actually just makes my anxiety worse. So, I’d rather be either just me and my husband or just nobody, just by myself, if I have to, without kids. It’s an extremely lonely process”
Temporality	Unknown present and future	“When I first found out, I was at work. The thing is my Dad had been sick too, really quite needing a lot of care. So, when I heard about my Mom I really was just overwhelmed. I didn’t know what to do”
		“So, I remember it was a really rough morning. I paid [the travel costs] and then I got over there, I think I got over there at midnight . . . I went upstairs to see [her son] and he was doing okay, and then I went downstairs to just get a coffee and pay for my parking and that would’ve been my 70 bucks gone . . . thankfully they have the Ronald McDonald room downstairs and they do usually do suppers and stuff like that, and there’s always snacks and stuff like that. So, that’s the first thing I do is I go downstairs and see what the plan is for dinner for them if I know I don’t have money for dinner . . . There’s no charge for it . . . it’s all free”

*Note*. OR = operating room

### Vulnerability in the Urban Center

Family members described feelings of vulnerability in the urban center where their relative was transferred, particularly if they were alone without family support. Feelings of loneliness, fear, and homesickness were prevalent. Compared with those familiar with the city, participants who were unfamiliar with the (transfer) city described fear with wayfinding, driving in the urban area, and being outside after dark. A mother who had limited financial resources available to her described this context as “terrifying” and wondered what she would eat because she had no money to purchase food. For family members who were older, being in the urban center also evoked feelings of vulnerability specific to their age. Thus, it was important for older participants to have family with them in the urban center and, without this family support, they would have been more frightened. An older wife stated “It wasn’t bad because I had my three boys in the [rented] house with me. But if I’d been there [alone], I wouldn’t be sleeping because I’d be nervous.” This highlights the embodied experience within an unfamiliar urban center.

### Feeling of Peace

Interspersed within the feelings of stress and fear were feelings of peace. Family members traveling to the urban center reported a sense of relief when they arrived safely at the urban center. Others expressed feelings of relief when they received notification that their critically ill family member arrived safely. Seeing their relative for the first time after the IFT evoked feelings of calm upon seeing that they were still alive. It was very difficult for family members to bear witness to their relative’s suffering and seeing their critically ill relative calm and at peace resulted in the family member also feeling at peace. Family members who maintained connection with family at home using virtual audio-visual means noted labile emotions that alternated between happiness and sadness.

Some family members attempted to gain control and, thus, a sense of peace through a variety of actions. A mother packed her van with everything she could think of that she and her son might need in the urban center prior to leaving home. A daughter and mother sought to actively perform personal care for their critically ill relatives and engage in care planning with the health care team. A son assumed the role of health care provider for the 4-hr drive home after his older adult father, who underwent neurological surgery 2 days previously, was discharged home. Active behaviors helped to reinstate a sense of control and calm their minds.

### Need to Protect

Family members experienced a wish to protect their critically ill relative, and at times other vulnerable members of their family, resulting in a sacrifice of their own needs. When a critically ill relative shared a room in the urban center with another patient who was agitated or had visitors perceived to be a risk to the well-being of their relative, family members would purposely take shifts sitting at the bedside for protective purposes. As well, for some family members, it was important to be present at the bedside to receive information from health care providers so that they could then make the best health care decisions for their critically ill relative. In some cases, participants described a concern that their relative might be frightened and by being at their bedside they could provide reassurances to them and reduce their fear. This desire to protect also extended to other members of the family who were perceived to be vulnerable. A son expressed an intrinsic need to ensure that his older adult mother had nutritious food and obtained sufficient rest while his father was critically ill. A friend of a critically ill patient also described her need to provide emotional support to the patient’s children who were far away from the urban center.

Family members who were not permitted to travel to the urban center because of COVID-19 interprovincial travel restrictions also experienced a desire to protect their critically ill relatives. A sister described how important it was for her to call the urban center frequently for updates and ask the nurses to tell her brother that she had called, so that he would not feel alone. Another sister, who was unable to be present when her brother was discharged from the urban center because of interprovincial travel restrictions, was displeased that she was not a part of the discharge teaching, which left her struggling to meet her brother’s needs such as dressing changes, medication adjustments, and safely increasing mobilization.

### Maintaining Responsibilities

Participants detailed numerous responsibilities to which they needed to attend and maintain during their relative’s critical illness and IFT to an urban center. Some participants described a multitude of competing demands both at home and the urban center. For instance, a wife explained how she had to frequently commute 4-hr home to manage her rental business, her husband’s construction business, care for their pets, and then return to the urban center to be with her husband. Other family members, both middle-aged and older, described situations where they were simultaneously providing care to ill siblings at home while also striving to be at their critically ill relative’s bedside in the urban center. Many participants indicated that they did not ask for help in managing these responsibilities, as they perceived this to be their role within their family. This role also included managing and organizing family members, updating concerned family members regarding their critically ill relative’s status, and acting as the primary decision maker. One daughter related “[it] took a while getting to sleep. I kept texting, catching people up.” Assuming these roles often resulted in family members forgoing their own rest and sleep, which compromised their own well-being.

### Supported by Health Care Providers

Family members identified numerous relationships that were perceived as supportive during their critically ill relative’s IFT to an urban center. Participants described connections with health care providers as important and necessary to help them cope with and understand the situation. Being welcomed into the critical care area and receiving up-to-date information was comforting, and family members identified that it was important for them to be able to provide information about their relative to the health care team; in doing so, they were able to contribute to the care of their relative. When family members trusted the health care team, it was easier for them to endure separations, such as during the transfer window or during surgical procedures. While family members expressed a need for information, many were hesitant to “bother” the nurses and doctors. An older adult wife of a critically ill patient described her experience stating, “a couple minutes to talk to you and tell you what’s going on then you’d be, you’d be happy and they wouldn’t have to feel like you’re hounding them for information. Which you don’t like to do.” Some family members described that they felt hesitant speaking with nurses in the urban center compared to nurses in their local hospital. When describing why they felt more comfortable speaking with critical care nurses at the local hospital, one participant, a middle-aged sister stated “I don’t know, it’s just different. Maybe it’s just me, maybe I feel different. We all live here in [home province] so it’s easier to talk here.”

### Navigating Relationships

Connections to family, friends, and strangers were perceived as positive and important during a relative’s IFT to an urban center. Many participants suggested that it was these connections that helped them to cope with and get through this experience. Support from family and friends included regular in-person or telephone contact, assistance with meeting basic needs, such as arranging accommodation and food, financial support if needing to pay for essentials in the urban center, wayfinding in the city, and, for some participants, driving them to the urban center when they felt uncomfortable driving far from home. Family members with relatives who lived in the city felt this to be additionally supportive because they would come to visit them and, in doing so, make the family member from the rural area comfortable enough to leave their critically ill relative’s bedside for a much-needed reprieve from the hospital. A daughter recalled “I knew I had people in [the city] . . . my son came right over and stayed with me [in the urban hospital] . . . it was really nice to have him there.” For family members who were alone in the city, a pervasive feeling of loneliness was described. Having other family members physically present with them in the city mitigated these feelings of loneliness.

For some participants, family and other responsibilities were viewed as contributing burden to their experience. A wife described feeling “let down” by family and friends that they expected to be available to support them during this experience. Relationships with family members that were perceived as challenging or difficult before their relative’s critical illness incident often were exacerbated during the IFT event and added stress. Some family members indicated that using social media was useful as a tool to keep in touch with friends and family, whereas others noted that this generated stress. Family members also remained deeply connected to their critically ill relative during their IFT to the urban center. Consistently, participants described a persistent need to be close to their relative, if not physically, then close in mind.

### Loss of Connection

Forced separation from their critically ill relative was noted to be distressing for family members. Family members frequently referred to the time during which their critically ill relative was in transit, a time when there was no means of communication between family members and the transport team caring for their relative. This transfer window lasted between 3 and 4 hr, and was described as intensely stressful, the hardest part of the event to endure. For some, the transfer occurred during adverse weather conditions, such as blizzard or dense fog; this extended the transfer time and increased the stress that family members faced. Family recalled this period as a time when they wondered if their relative was still alive, if they made it to the hospital, and what would they discover when they themselves arrived at the urban hospital. A daughter who was provided the transporting paramedic’s mobile phone number indicated that the ongoing communication during the transfer was invaluable to her; she was not sure how she would have been able to cope without this communication.

Some participants requested to accompany their critically ill relative in the land or air ambulance but were denied this option by the transfer team. Despite a persistent wish to be close to their critically ill relative, family members often described a reluctance to interrupt or potentially obstruct the work of health care providers. This reluctance extended to a hesitation of family members to telephone the urban center for updates because they did not want to be “bothering the nurses. They’re looking after the patients.” Some participants described feeling a sense of a lack of privilege to be physically present in the critical care area and others noted that they did not want to “be in the way” of the doctors and nurses who were looking after their critically ill relative. A tension existed whereby family members resisted action to meet their need to be physically close to their critically ill relative.

### Pulling Patient and Family Close

As explained, the meaning of the experience of a critically ill relative’s IFT to an urban center is influenced in a relational way through family members seeking to maintain a close connection with their relative. However, despite family members’ need to be physically close to their critically ill relative, forced separations did occur and were endured by participants. In these situations, family members described how they pulled the patient close to them in their mind during the transfer and while they were admitted to the urban center. For family members who were unable to travel to the urban center, they kept their critically ill relative close in mind through telephone contact or by trying to anticipate their needs. Family members who traveled to the urban center also described how they pulled family members at home close to them in mind through frequent telephone calls to share information and experiences. At times, these telephone calls were particularly difficult. For example, when sharing bad news with a family member at home, a close friend stated “it is different when you are talking to him and you can’t see his face, you can’t hug him, you can’t do anything.” In this way, being physically apart from family members influenced the meaning of delivering difficult news to others who were not present at the urban center.

Despite this prevalent need to be close to their critically ill relative and other family members, some participants described an intermittent need to push away from the physical space of their relative’s bedside. In doing so, participants described gaining some much-needed space for themselves. However, to feel comfortable to momentarily part from their critically ill relative, family members indicated a need to trust that the health care providers would watch over their relative while away from the bedside. Alternatively, if other family members were present in the urban center, their presence at the bedside also provided a sufficient level of comfort for the family member to take a break from the bedside. Thus, participants sought a balance between closeness to their family member and distance from the hospital.

### Home and Away

Participants often perceived the IFT for their critically ill relative as a positive event where they would receive the advanced care that they required. However, some family members expressed a fear for their relative’s safety during the transfer by ambulance or helicopter. This was especially experienced by family members if the transfer occurred during adverse weather conditions, such as fog or blizzard conditions. When family members arrived in the city, those who were unfamiliar with the city or city driving experienced feelings of stress and insecurity. Upon arrival at the hospital, some family members experienced difficulty finding a place to park and then, when entering the hospital, were frustrated and stressed with their inability to locate their critically ill family member. A friend and next of kin of a critically ill patient describes

we had to find our way up to where neurosurgery is ‘cause I’ve never been in that hospital before . . . I just wanted to get to neurosurgery and it is such a big hospital . . . we did get lost a couple of times.

The environment of the urban hospital and critical care unit was described as busy, foreign, shocking, and sterile by some.

The concept of *home* represented rest, comfort, and support. Some participants described *home* as a geographic place or area that represented normality and peace. Other participants described *home* not in geographic terms, but rather relative to their network of family and friends that surrounded them. For these individuals, they did not express a loss of *home* during the IFT; rather, their *home* came with them to the urban center in the form of their friends and family members. While in the urban center, participants pulled close elements of *home*. They tried to make connections with health care providers, connect with family who lived in the city, and held close items that reminded them of home. Having family with them resulted in the hotel room feeling more like *home* than a hotel. Participants also described the use of technology to connect with family at home to make the urban center feel more *home*-like. *Home* was also close in mind for some participants because they described feeling torn between responsibilities at both the urban center and at home, which was the case for participants who left work to travel to the urban center or had young families at home to care for. Other participants indicated that they had no concerns with responsibilities at home, allowing them to be more settled while away.

### Unknown Present and Future

With the sudden onset of a relative’s critical illness and subsequent IFT to an urban center for advanced critical care services, family members experienced a sudden shift in their present reality and the future became unknown. While traveling to the urban center, participants described the phenomenon of not knowing what their present reality was for several hours and if their relative had survived the transfer to the urban center. A sister described, “we just wanted to get there. We were just hoping and praying that there’s nothing happening.”

The onset of the COVID-19 pandemic added additional uncertainty of the present context for many participants; family members described the backdrop of the rapidly evolving pandemic as contributing additional insecurity and stress to their experience of a critically ill relative’s IFT to an urban center. Evolving public health guidelines ranged from a requirement to self-isolate for 14 days after return from interprovincial travel (Government of Canada, 2021) to the tightening of restrictions such that no travel was permitted beyond that which was necessary for urgent health care (Government of Canada, 2021). For family members of critically ill patients undergoing IFT who were permitted to travel, a choice had to be made to either follow their critically ill relative to the urban center (and then self-isolate upon return to their home province) or remain in their home province during their relative’s IFT, so that they could then tend to their relative upon their return home, without the restriction of self-isolation. For participants who experienced a relative’s IFT during a time when no travel was permitted, the wait at home for notification that their relative arrived safely at the urban center was a time of intense stress and anxiety. The dynamic context of the pandemic and associated public health guidelines and restrictions added additional stressors to these family members and additionally contributed to the sense of the unknown present and future.

The sense of a previously, predictable future suddenly becoming unknown influenced the meaning of this experience for family members. Participants described a “wait and see game” where the future was unknown and could not be known for some time. For example, not knowing how long they would be at the urban center, if their relative would sustain permanent damage from the event, or if they would survive were pervasive thoughts. Some participants noted that they tried to pass this time or consciously will it to move faster. For participants who were already burdened with managing care needs of other family members, this unknown future of an unexpectedly critically ill relative was overwhelming.

Uncertainty was also tied to financial concerns. Those participants who were in a strong financial position did not have monetary concerns while spending time in the urban center with their critically ill relative. Other participants described worrying about costs adding up over time, particularly if they needed to remain in the city for a prolonged period. Family members who described their financial situation as precarious expressed considerable stress with regards to the cost of travel to the urban center along with the costs of accommodation and food that they subsequently incurred. This uncertainty associated with the suddenly unknown present and future was pervasively influential to the experiences of participants.

## Discussion

According to Munhall (2012), through understanding individual meanings of experience, nurses may better individualize the care provided to patients and families, thus reducing negative outcomes and enhancing well-being. To the best of our knowledge, this research is the first to explore the context-specific meanings of this experience for rural Canadians. In this manuscript, findings of the present study centered around family relationships are described with the aim of increasing awareness of nurses as to what meanings are possible for family members from rural areas. The increased understanding gleaned from the findings can support family theory and intervention development, thereby, improving the care of this unique population.

Through consideration of the participants’ experiences of a family member’s IFT for advanced critical care services, both challenging and beneficial aspects of experience were identified. As with previous studies, family members in the present study experienced concerns about managing responsibilities at home while away at the urban center, fear and uncertainty related to traveling to an unfamiliar city and hospital, worry that something would happen to their relative while enroute to the urban center, and, for some, concern related to how they would manage financially (Johnson, 1999; Karlsson et al., 2020; Mackie et al., 2014). These findings also overlap with transfer experiences for other health-related indications, such as pregnancy-related conditions (Woodhart et al., 2018) or cancer-related care (Chua et al., 2022). These IFTs also can involve dangerous travel conditions for the patient (Woodhart et al., 2018), competing demands for the family members to managing responsibilities at home, and mounting financial costs for the family to bear (Chua et al., 2022; Woodhart et al., 2018).

Karlsson et al. (2020) and Mackie et al. (2014) indicated that receiving information from health care providers that is honest and complete is beneficial for family members and the absence of this is distressing, which was an experience echoed by participants in the present study. However, despite this need for information, participants in the present study often described an aversion or reticence to communicating with health care providers or even being present in the critical care area for fear of “bothering” health care providers. This is similar to the finding by Bell et al. (2018) who found that family members chose not to voice concerns to health care providers because they felt that they did not have the required knowledge or understanding of their relative’s health care situation, or they perceived the team to be too busy. In addition to this, one participant of the present study noted she was less comfortable speaking with nurses in the urban center compared with nurses in the rural center; with nurses in the rural hospital being from and living in the same geographical location as her, it felt easier to talk to these individuals. It has been noted in previous work that family members generally are reluctant to communicate concerns with intensive care unit (ICU) health care providers, thereby suffering in silence (Bell et al., 2018; Burns et al., 2018; Karlsson et al., 2020). However, the discomfort of family members from rural areas, specifically when communicating with nurses in urban centers, has not previously been noted. The findings of this study raise the question about whether the variable of rurality increases hesitation and discomfort of communicating with ICU health care providers. According to Slama (2004), individuals living in rural areas share a culture with characteristics, including a respect for perceived authority, self-sacrificing personal needs in preference for the needs of others, and an intrinsic difficulty in communicating concerns with individuals unknown to them, such as nurses in the urban center.

In addition to the consideration of rurality as culture, is the consideration of how health literacy or the degree to which individuals successfully interact with health care systems, may be influential. It has been noted that health literacy is an area of health concern in rural Canada (Gillis & Sears, 2012). This understanding of rurality as a distinct culture (Slama, 2004; Smalley & Warren, 2012) and its resultant influence on the meanings of experience for family members from rural areas are an important consideration. As an ICU nurse for more than 20 years, the first author has borne witness to numerous nurses questioning why some family members of critically ill patients do not call for updates on their relatives or do not come in to visit them. It is important to remember that each individual encountered in the health care environment has a unique history, experience, and culture that has shaped their life-world and, thus, their perceptions and actions (Munhall, 2012).

Health care inequities exist between individuals residing in rural and urban areas (Bourke et al., 2012; Nichols et al., 2020). Contributing to these inequities is the regionalization of health care (Nichols et al., 2020) and urban centric health services and policies (Bourke et al., 2012). Consequently, individuals in rural areas have unique health care experiences distinct from individuals in urban areas. For example, as in the present study, previous work has noted the financial burden experienced by family members when obtaining accommodation in the urban center, far from their home (Lewis et al., 2020; Mackie et al., 2014; Woodhart et al., 2018). Another experience unique to family members from rural areas is the transfer window when the critically ill relative is in transit from the rural hospital to the urban receiving hospital; a time lasting several hours in which there is an absence of communication between the family and the transfer team. As noted in the present study, family members described this as the most difficult part of the experience to endure; thus, highlighting the importance of access to and connection with the critically ill relative during the transfer window, an experience not endured by family members from urban areas. However, the importance of communication between family and the transfer team during the IFT window for advanced critical care services has not previously been described. Additional study is required to explore this form of support for family members from rural areas whose critically ill relative undergoes IFT to an urban center.

Family members in the present study described a persistent need to be close to their critically ill relative prior to transfer, during transfer, and then in the urban receiving center, a finding supported by previous studies (Karlsson et al., 2020; Mackie et al., 2014). For those family members that were not permitted to travel in any way to the urban center because of COVID-19 interprovincial travel restrictions, it was particularly important for them to be present in their local emergency department to say goodbye to their critically ill relative. Then, fearing that their relative may feel lonely in the urban center, family members called the urban center frequently to leave messages with the nurses to give to their relative. The experience of loneliness has been described in patients who are transferred from rural to urban settings for cancer care (Chua et al., 2022) and pregnancy-related care (Lewis et al., 2020). Several participants asked the transport team if they could travel with their relative but were refused this option. While it has been supported that family members may safely accompany critically ill relatives, family members often have been denied this opportunity because of the personal assumptions of the transport team (Brown et al., 1998). More recently, Plante et al. (2020) found that while 74% of pediatric critical care transport team members appreciate that accompanying the critically ill child during IFT is beneficial for family members, personal assumptions related to risk persist. These assumptions include concern about limited space within the ambulance, that the family member may panic during IFT and subsequently interfere with the care of the patient, the family member may experience negative psychological or emotional consequences related to the experience, and the presence of the family member may increase the anxiety of the transport team during the IFT (Plante et al., 2020).

The context of the global COVID-19 pandemic resulted in several unique findings. At present, the experience of family members from rural areas who are not able to travel to an urban center when a critically ill relative undergoes an IFT is poorly understood in the literature. Although not the initial aim of this study, the context of the COVID-19 pandemic and associated travel restrictions can provide insights into other experiences where family members are unable to travel to an urban center. As noted by Mackie et al. (2014), and as evident in the findings of this study, being present and involved at a critically ill relative’s bedside may assist family members in regaining a sense of control during an experience that often feels out of control. Similarly, within the context of a rural to urban IFT for other indications, such as pregnancy-related conditions, it is suggested that supporting family members to remain close to hospitalized relatives may reduce their distress (Woodhart et al., 2018). Thus, denying presence or involvement has the potential to negatively impact the well-being of family members from rural areas. Through the meanings expressed by those denied the option to travel to the urban center in the present study, an initial understanding of this experience and its challenges is offered.

For those who could travel to the urban center, family members frequently remained at their relative’s bedside or in the waiting room of the critical care unit. When choosing accommodation, a priority for many family members was to remain close to their relative and, as such, those close to the hospital were frequently chosen despite these typically being more expensive. These findings are similar to previous work describing this need for accommodation close to their relative which results in a financial burden for family members of critically ill patients (Mackie et al., 2014) and patients who require IFT for pregnancy-related care (Lewis et al., 2020; Woodhart et al., 2018).

Family support during a critically ill relative’s IFT to an urban center was described by participants as very important. When participants were accompanied by other family members to the urban center and supported by these individuals during their stay in the city, these family members provided practical support, such as arranging accommodation, organizing meals, and taking turns at the patient’s bedside. When describing their experiences, family members often noted that they could not have coped without this level of family support, a finding echoed by Mackie et al. (2014). The present study supports Mackie et al. and Johnson’s (1999) finding that separation of a family member from their network of support at home is detrimental and exacerbates the degree of anxiety experienced by family members from rural areas. However, it must be noted that some family members in the present study described negative experiences including behaviors by extended family members that were perceived to be selfish or in some way burdensome. Family conflict was also described as detrimental to well-being by Mackie et al. and can be significant enough to risk the functioning of the family.

Similar to findings reported by Karlsson et al. (2020), participants of this study experienced a persistent longing for home. However, *home*, as longed for by these family members, encompasses more than the physical home; rather it includes both past and present family connections, routines, familiar items, memories, and a sense of normality. Participants described a sense of *home* present with them in the urban center through the presence of other family members or familiar items, such as books or their vehicle. Longing for home was portrayed as the family member pulling close a sense of *home* through telephone or video calls, reminiscing about *home*, and wishing for their critically ill relative to recover and be discharged *home*, a return to normal. While family members have indicated that the transfer of their relative to a hospital closer to home is positive and a shift toward a sense of normality (Karlsson et al., 2020), the concept of *home* as conveyed by participants in the present study has not previously been noted. This novel finding is important because it suggests that *home* may be more mobile than previously perceived by critical care nurses working in an urban setting. As such, critical care nurses are in a unique position to encourage and support family members from rural areas to draw on elements of *home* beyond the physical or geographical and pull these close to provide comfort while in the urban center.

### Strengths and Limitations

This study had several strengths. In addition to the phenomenological expertise by some co-authors, the first author undertook extensive reading and writing to gain an increased understanding of the philosophy of phenomenology, a critical step in completing a trustworthy phenomenological study (Annells, 1996; Koch, 1995; Munhall, 2012). Another strength relates to the first author’s 20 years of experience as a critical care nurse in both rural and urban centers. This practical knowledge of IFTs, considered within the approach of *unknowing*, facilitated the conversational nature and search for meaning during participant interviews that is encouraged by Munhall (1994, 2012) to promote rigor. Another strength of this study is related to the uniqueness of the context within which it was set, as there are no other studies in this area which were undertaken in North America. Canada is a geographically vast country with diversity of culture and experiences. Thus, findings of the present study reveal meanings of this experience for family members within the Atlantic Canadian context. While these meanings may be different from those experienced by family members in different rural locations within Canada, they have potential to be transferable to other rural to urban transfer experiences beyond the study context. Through understanding the unique realities of patients, nurses can individualize care and, thus, enhance each nurse–patient relationship. In this way, the use of phenomenology is viewed as a strength in building nursing knowledge.

The present study encountered several limitations. Individual narratives were offered to respective participants to review to ensure that the meaning of their experience was accurately captured. Ten participants confirmed that the narrative of their experience was acceptable and required no changes. One participant declined to review their narrative; however, there is no reason to believe that it was inconsistent with their experiences. In addition, potential gaps in understanding of experience remain, as recruitment of men was limited to two participants, a noted challenge in many studies (Markanday et al., 2013). Also, none of the participants in the present study experienced the death of their critically ill relative. As such, the meanings within these contexts remain poorly understood.

## Implications and Recommendations for Family Nursing Practice and Research

Qualitative research methodologies, such as interpretive phenomenology, generate findings that move nurses to reconsider previously held assumptions and become sensitized to the unique individual experiences of each patient or family they care for (Munhall, 2012). Munhall’s (1994, 2012) method of interpretive phenomenology extends beyond the generation of findings to culminate in the writing of an overarching narrative or story. Narratives are a powerful means of communicating new insights gained from an inquiry (Burns & Peacock, 2019; van Manen, 2014). The narrative makes explicit, the similarities and differences of experience that are possible, and is directly influenced by an individual’s unique cultural, social, and personal history (Munhall, 2012). In doing so, this narrative offers insight into previously underappreciated individual experiences and, thus, inspires reflection and practice change (Crowther et al., 2017; Munhall, 2012; van Manen, 2007). As such, nursing care is delivered in an individualized manner rather than a standardized care approach informed by evidence from “aggregates of individuals or the non-existent person” (Munhall, 2012, p. 170).

As articulated by Munhall (2012), a phenomenological study should lay the foundation for a “call to action” to change practice to better care for patients and families (p. 168). The findings of this study as reported in this paper and in an overarching narrative have implications for nursing practice in both urban and rural settings. However, while differences in experiences between family members in rural and urban areas are identified in the present study, it must be emphasized that uniqueness in experiences for families within the rural context are also inevitable.

Foremost, family members have an urgent need to remain close to their critically ill relative both during transfer and at the urban center. Some family members describe the separation during transport as the most difficult part of the entire experience. Transport teams should make every effort to accommodate a family member accompanying their critically ill relative during transfer to the urban center. If this is not possible, transport teams should consider providing the next-of-kin family member with a contact number that can be used to communicate during the transfer; having access to contact the transport team may mitigate the intense stress family members experience during this part of the event.

In addition, nurses in rural settings have an opportunity to support family members from rural areas during this event by ensuring they feel comfortable at the critically ill patient’s bedside and advocating for the family member to accompany the patient in the land or air ambulance. Also, nurses in these rural settings can assist family members by providing clear information about the process of IFT, directions to the urban center, and accommodation choices that are in close proximity to the receiving hospital. Nurses in rural areas should also assess for potential financial concerns that these family members may have and collaborate with other members of the interdisciplinary health care team and available resources to support families financially during this time.

With insights revealed by this study, nurses in urban settings can better appreciate the unique challenges experienced by families from rural areas, such as a loss of family support, need for information, and a need to be close to their critically ill relative. With this knowledge, nurses in areas may appreciate the additional emotional, psychological, social, and practical supports required by these family members who often remain silent in self-sacrifice. Thus, rather than assuming silence as indicative of an absence of need, the nurse in the urban setting can strategically connect and interact with family members from rural areas to elicit previously concealed care needs.

While the present study supports previous work and extends the understanding of this phenomenon, more research is needed. Interventions using communication technology to enhance a sense of closeness to critically ill relatives during IFT need to be explored and evaluated. In addition, future studies are required to understand the meaning of this experience for family members whose critically ill relative does not survive the critical illness event.

## Conclusion

This study reveals the meaning of the lived experiences of family members from rural areas whose relative undergoes an IFT to an urban tertiary center for advanced critical care services. This manuscript explicitly focuses on findings related to the impact of these lived experiences on family relationships. Accessing these meanings through the lenses of the four life-worlds, novel findings included the family members’ sense of *home* while in the urban center and the intensely stressful time of the transfer window. Family members from rural areas have unique needs and experiences that can add additional burdens to an experience like IFT that is already intensely stressful. With this enhanced understanding and appreciation of the many possible meanings that are context-specific and unique, nurses in both urban and rural settings can more effectively communicate with, understand, and support family members from rural areas during this experience.
